# Landscape of snake’ sex chromosomes evolution spanning 85 MYR reveals ancestry of sequences despite distinct evolutionary trajectories

**DOI:** 10.1038/s41598-020-69349-5

**Published:** 2020-07-27

**Authors:** Patrik F. Viana, Tariq Ezaz, Marcelo de Bello Cioffi, Thomas Liehr, Ahmed Al-Rikabi, Leonardo G. Goll, Anderson M. Rocha, Eliana Feldberg

**Affiliations:** 1 Coordenação de Biodiversidade, Laboratory of Animal Genetics, Instituto Nacional de Pesquisas da Amazônia, Av. André Araújo 2936, Petrópolis, Manaus, AM 69067-375 Brazil; 2 Institute for Applied Ecology, Faculty of Science and Technology, University of Canberra, ACT 12, Canberra, 2616 Australia; 3 Departamento de Genética e Evolução, Universidade Federal de São Carlos, São Carlos, SP Brazil; 4 Institute of Human Genetics, University Hospital Jena, Am Klinikum 1, 07747 Jena, Germany; 5 Faculdade Cathedral, Laboratório de Zoologia Aplicada de Vertebrados Terrestres E Aquáticos, Av. Luis Canuto Chaves 293, Boa Vista, RR Brazil

**Keywords:** Cell biology, Evolution, Genetics

## Abstract

Most of snakes exhibit a ZZ/ZW sex chromosome system, with different stages of degeneration. However, undifferentiated sex chromosomes and unique Y sex-linked markers, suggest that an XY system has also evolved in ancestral lineages. Comparative cytogenetic mappings revealed that several genes share ancestry among X, Y and Z chromosomes, implying that XY and ZW may have undergone transitions during serpent’s evolution. In this study, we performed a comparative cytogenetic analysis to identify homologies of sex chromosomes across ancestral (Henophidia) and more recent (Caenophidia) snakes. Our analysis suggests that, despite ~ 85 myr of independent evolution, henophidians and caenophidians retained conserved synteny over much of their genomes. However, our findings allowed us to discover that ancestral and recent lineages of snakes do not share the same sex chromosome and followed distinct pathways for sex chromosomes evolution.

## Introduction

Non-avian reptiles’ evolution is dynamic and remarkably diverse, especially in their sex-determining strategies, making them an interesting group to investigate the evolutionary trends of sex chromosome evolution^1–5^. An array of different strategies of sex determination have been reported in different vertebrate lineages^6–9^. However, non-avian reptiles display the most diversities, including TSD (Temperature-dependent Sex Determination), GSD (Genotypic Sex Determination) and GSD with the influence of temperature (reviewed in^3, 5^). In addition, variants of major mode of sex chromosome systems (XY, ZW and multiple chromosome systems) are frequently present among reptile clades, even within sister clades as well as within allopatric populations of the same species^10–16^. Moreover, frequent transitions between modes of sex-determining mechanisms (e.g. TSD–GSD–TSD) are also evident^1,12,15,17,18^. Such variabilities highlight the complexity of sex chromosome evolutionary history in this group. Besides, non-avian reptiles are the sole vertebrate group where master sex-determining genes are yet to be discovered, although some candidates have been suggested for few groups such as geckos, agamids, varanids and testudines^19–22^. Due to these enormous diversities in modes of sex determination and sex chromosomes across reptilian lineages, it is therefore not surprising that sex determination and sex chromosome evolution in non-avian reptile remains as a matter of much discussion and debate over decades.

Among non-avian reptiles, the majority of the snakes karyotyped so far exhibit a ZZ/ZW sex chromosome system, with different stages of evolutionary degeneration or amplification of W chromosomes^23–25^. However, undifferentiated sex chromosomes (and more recently unique Y sex-linked markers, suggesting an XY male heterogametic system) have also been reported in some genera such as in *Python* and *Boa*^26^. This implies that at least two transitions involving XY and ZW and independent turnovers of these sex chromosome systems may have occurred in Pythonoidea and Booidea superfamilies.

The gene content of both the Z and the W chromosomes are thought to be relatively conserved in snakes^24,27,28^. Still, the high variability of W chromosome (regarding morphology and/or gene content) in major clades suggests a remarkable role of repetitive sequences accumulation in their architecture and evolution^24,25,29^. Unlike the W, the Z chromosomes are thought to be more stable among Serpentes lineages, and non-drastic shifts in morphology have been reported in different snake clades^23–25,30^.

Although suggested as having an independent origin, the homomorphic XX/XY chromosomes in *Boa* and *Python* are yet to be characterized by molecular cytogenetic techniques. Thus, their relationships and transitions among both homomorphic and heteromorphic ZZ/ZW in Pythonoidea and Booidea superfamilies^31–33^ still remain unanswered. In the amazonian red-tailed *Boa constrictor* (formally *Boa constrictor constrictor*), which has 2n = 36 chromosomes, the fourth chromosomal pair is thought to represent the putative sex pair, which would be a typical feature for henophidians (i.e. a former superfamily of the suborder Serpentes, which harbors boas, pythons and other old lineages of snakes, usually referred as "*Primitive Snakes*")^31^. In addition, two different classes of sequences, the *PBI-MspI* and *EQU-BamHI-4* (*EQU-BamHI-4* being putatively reported as sex-linked^27,35^) have been identified on the 4th homomorphic pair of *Boa constrictor* females, therefore, being identified as the sex pair in these studies^34,35^. Similarly, in most of the pit vipers, rattlesnakes and colubrids, the 4th chromosomal pair also represent the sex chromosomes^24,25,36–38^, even when homomorphic, as already identified through accumulation of *Bkm* repeats in some species^39,40^, suggesting a conserved trend for sex chromosome evolution in Snakes.

Homomorphic sex chromosomes are frequently observed among non-avian reptiles. In the Serpentes suborder, for instance, they are found in major clades of henophidian and caenophidian species (i.e. Caenophidia is a monophyletic group that contains over 80% of all the extant species of snakes, commonly referred as “*Advanced Snakes*”)^30,31,39,41^. On the other hand, well-differentiated sex chromosomes are more common in the more recently diversified groups of snakes, the advanced lineages^25^, but also present in the former groups of snakes as Typhlopoidea and Booidea^32,42^. The molecular and cytogenetic mechanisms of evolution of homomorphic sex chromosomes in snakes have not been the subject of rigorous studies compared to well-differentiated ones. Therefore, many homomorphic or micro W or Y sex chromosomes in the ancestral lineages of snakes remained undetected. Serpentes was thought to have a well-stable sex chromosome system and, despite different levels of W degeneration, as well the occurrence of multiple sex chromosomes (e.g. Z1Z2/Z1Z2W and Z/W1W2), only ZW system had been described until recently^43,44^. Indeed, the arise of a putative and independently evolved XY sex chromosome system in Boidae and Phytonidae raised questions regarding the cytogenetic and molecular mechanisms involving the evolution of sex chromosomes in snakes. Why did snakes independently evolve a new and homomorphic sex chromosome system solely in ancestral lineages? Did snakes retain homology of XY and ZW chromosomes along the Serpentes’ evolution owing to some evolutionary advantage conferred by the shift and transitions between these systems? Why has the XY sex chromosome system been lost in the more advanced lineages?

Application of molecular cytogenetic tools, such as chromosomal mapping using Bacterial Artificial Chromosomes (BAC-FISH) and comparative genomic hybridization (CGH) have been instrumental in overcoming limitations in identification of undifferentiated or cryptic sex chromosomes in several vertebrate groups such as fishes^45–47^, amphibians^48,49^, and reptiles^20,50,51^. In this study, we aimed toward understanding the relationship between the homomorphic XY and heteromorphic ZW chromosomes found in some ancestral (henophidians) and more recent (caenophidians) snakes. For that, we performed an extensive comparative analysis of the amazonian red-tailed boa (*Boa constrictor constrictor*) chromosomes (homomorphic XY) through cross-species comparisons using whole genomic DNA (gDNA) from several caenophidian species with varying degrees of the ZW sex chromosomes differentiation. We also performed *WCP* (Whole Chromosomal Painting) of a highly degenerated W sex chromosome and mapped BACs specific for several genes on *Boa constrictor* chromosomes. We identified chromosome homologies of sequences for all analyzed species, however, with different patterns of accumulation, which enabled us to infer the relationship and landscape of snake’ sex chromosomes evolution spanning 85MYR.

## Results

### Chromosome painting with amazonian pit-viper (*Bothrops atrox*) W paints and cross-species chromosome painting to *Boa constrictor* chromosomes

**Figure 1:**
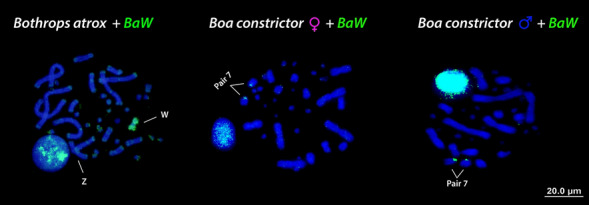
Chromosomal painting using derived probes of a highly heterochromatic and degenerated W chromosome from the amazonian pit viper (*Bothrops atrox*). The W probes (*BaW*) was used on the *B. atrox* chromosome spreads as control, showing large hybridized segments on the W chromosome. In the *Boa constrictor*, the *BaW* probe showed signals on the 7th pair. ISIS software was used for microphotography and analyzing images.

The isolated W chromosome probe of *B. atrox* (*BaW*) was amplified and the homology was tested onto metaphase spreads of the same species (Fig. 1). We carried out cross-species chromosome painting using *BaW* probe to metaphase spreads of male and female of *B. constrictor* in order to test for the homology of the sex chromosomes between Henophidia and Caenophidia. The *BaW* probe hybridized completely on a small metacentric (W chromosome/pair 4) of *Bothrops atrox* (Fig. 1). However, in male and female *B. constrictor* metaphase spreads the *BaW* probe showed faint hybridization signals on microchromosomes and on the centromeric position of the 7th pair (Fig. 1), with no differences between males and females.

### Comparative genomic hybridization

**Figure 2:**
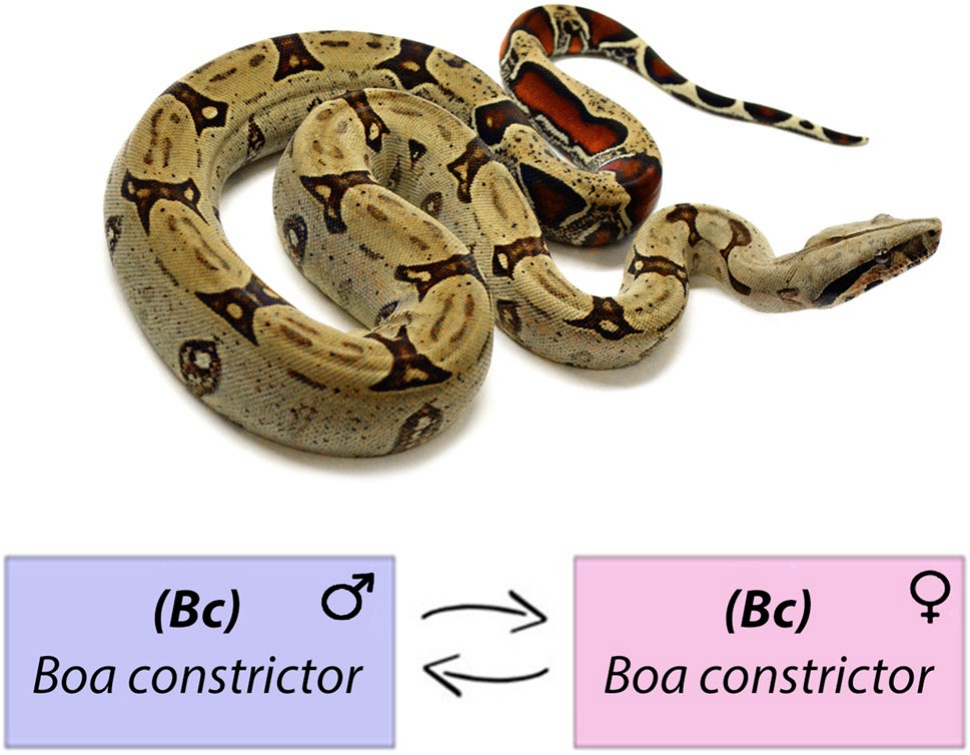
The experimental design used in this study where gDNA of male and females of *Boa constrictor* (***Bc***) were used for hybridization against male and female chromosomal background of *Boa constrictor* (***Bc***).

**Figure 3:**
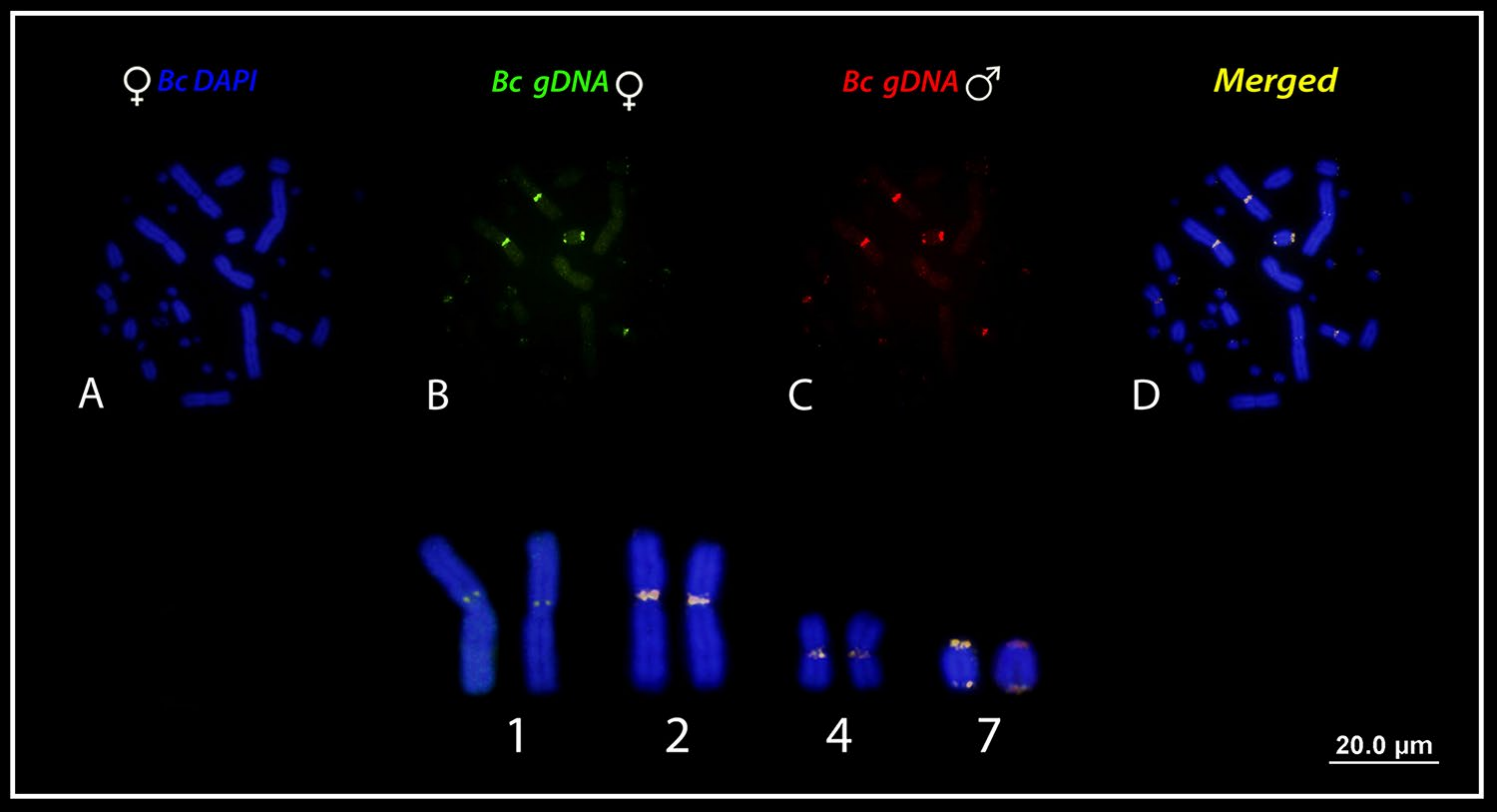
CGH comparison between *Bc* (*Boa constrictor*) male and female showing only shared sequences, mainly on the chromosome pairs 1, 2, 4 and 7. Microchromosomes showed faint signals, with no sex-specific pattern. ISIS software was used for microphotography and analyzing images, Bar = 20 µm.

Comparison between male and female gDNAs of *Boa constrictor* (Fig. 2), produced intense and faint hybridization signals on macrochromosomes and microchromosomes, respectively (Fig. 3), co-located with C-banded regions as previously reported^31^. Small-shared signals were observed on the pericentric regions of the *p* arms of chromosome pair 1, whereas strong bright signals were observed on the centromere of chromosome pairs 2 and 4 and on the telomeric position of chromosome pair 7. Male or female-specific hybridization signals were neither detected in *B. constrictor *male and female nor on the homomorphic sex chromosomes (putatively the chromosome pair 4) (Fig. 3).

**Figure 4:**
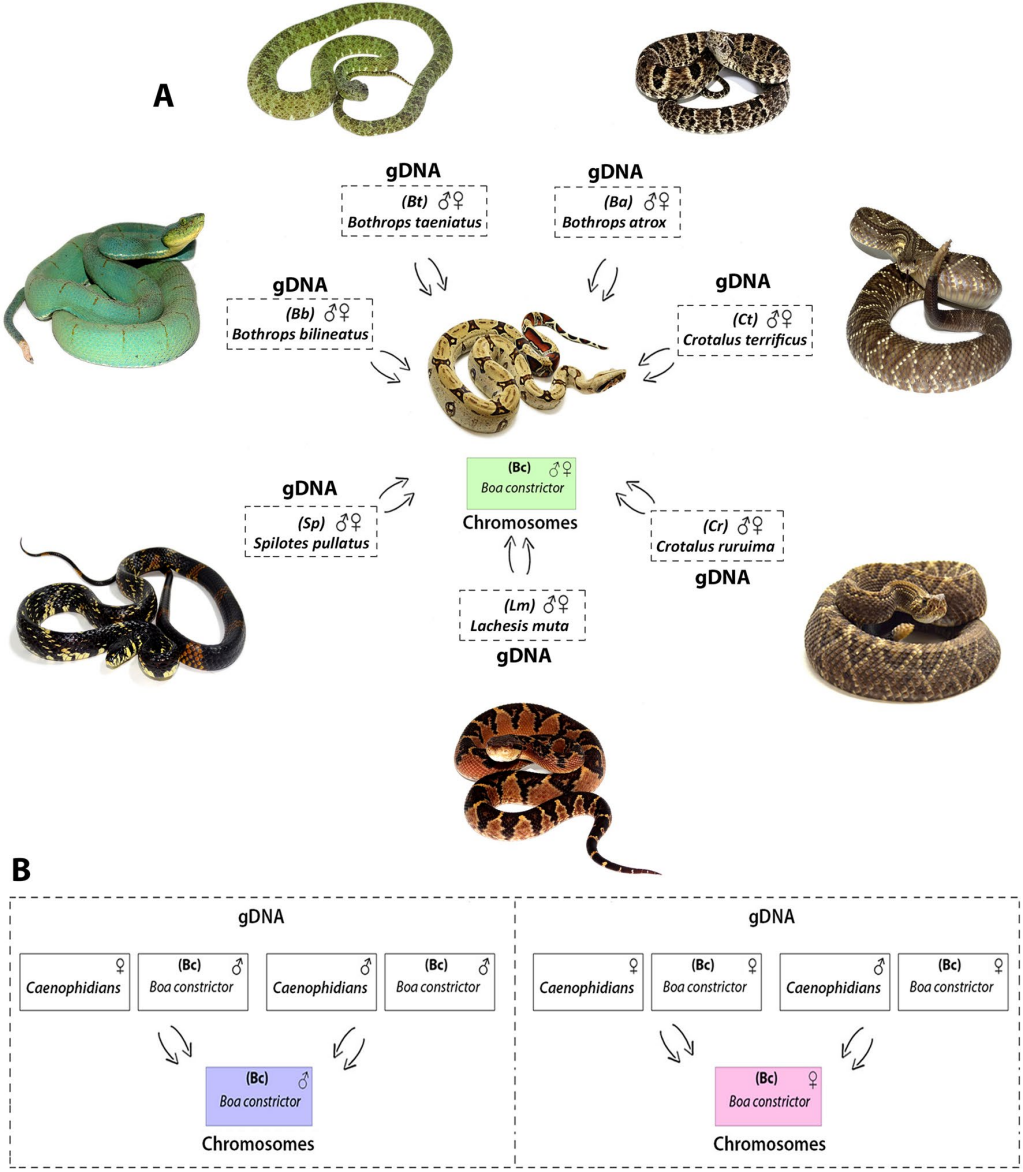
The second set of experiments, (**A**) male- and female-derived gDNAs of *Bothrops bilineatus* (***Bb***)*; Bothrops taeniatus* (***Bt***) *Bothrops atrox* (***Ba***)*; Lachesis muta* = (***Lm***)*; Crotalus terrificus* (***Ct***)*; Crotalus ruruima* (***Cr***) and *Spilotes pullatus* (***Sp***) were used for hybridization against male and female chromosomal background of *Boa constrictor* (***Bc***). In the (**B**) an example of how the second set of experiments was conducted, where the gDNA of male and female of caenophidians were used together the gDNA of boas male and females and hybridized on the chromosomes of *Boa* male and female. Pictures of the caenophidians by Ayrton Costa.

**Figure 5:**
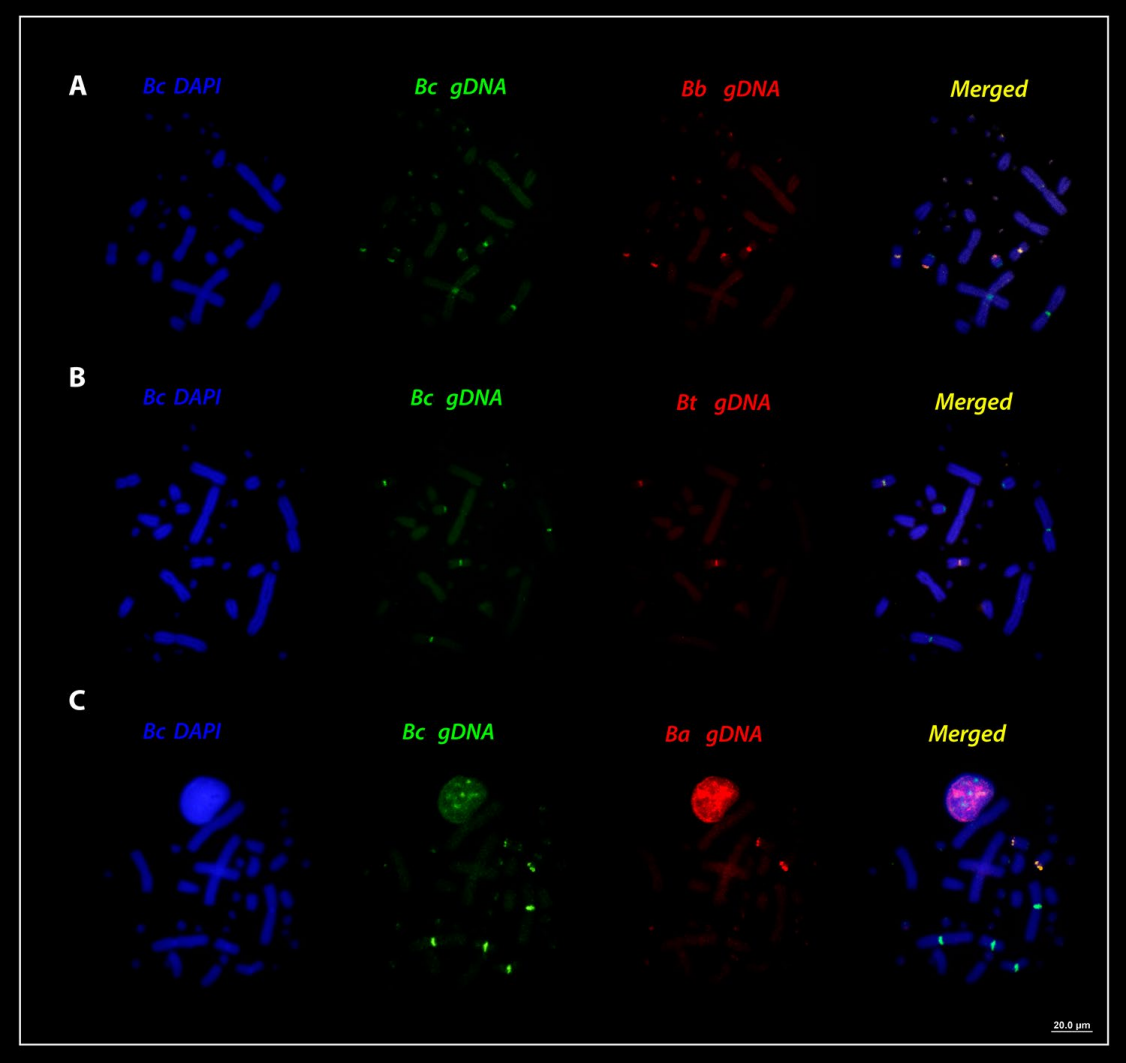
Cross-species comparisons between *Bc* (*Boa constrictor*) chromosomes and caenophidians gDNA. The cross-species hybridization (**A**–**C**) the *Bc* gDNA is in green and *Bothrops bilineatus*, *Bothrops taeniatus* and *Bothrops atrox* (*Bb*, *Bt,* and *Ba*) in red. ISIS software was used for microphotography and analyzing images, Bar = 20 µm.

**Figure 6:**
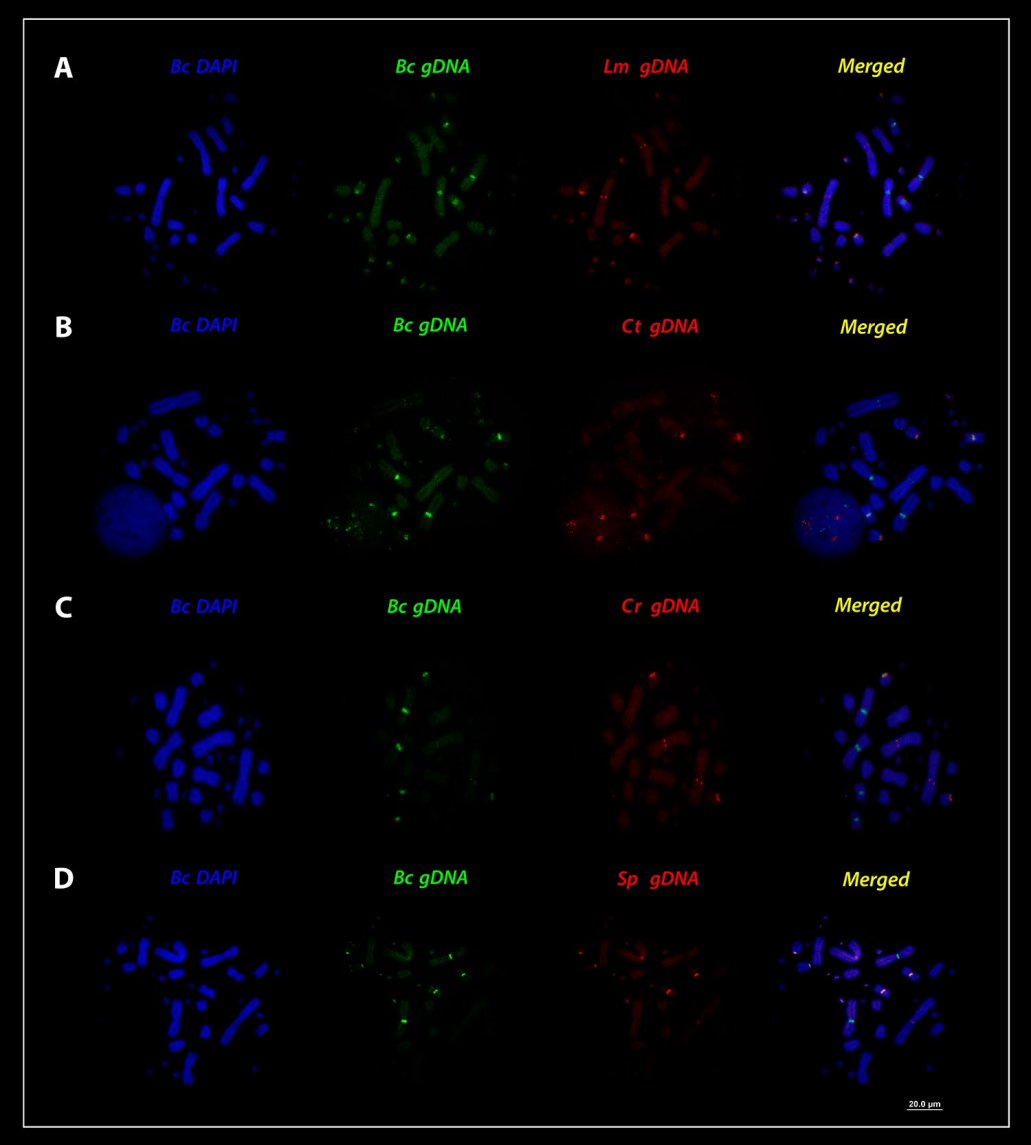
Cross-species comparisons among *Bc* (*Boa constrictor*) chromosomes (green) and caenophidians gDNA (red). (**A**) *Lachesis muta*, (**B**) *Crotalus terrificus*, (**C**) *Crotalus ruruima*, (**D**) *Spilotes pullatus* (*Lm*, *Ct*, *Cr,* and *Sp* respectively). ISIS software was used for microphotography and analyzing images, Bar = 20 µm.

Interspecific hybridization among henophidian (*Boa constrictor*—red-tailed boa) (ancestral lineage XY) and caenophidian (advanced lineages ZW) (pitvipers—*Bothrops*, bushmaster—*Lachesis*, rattle snakes—*Crotalus* and puffer snake—*Spilotes*) species (Fig. 4a, b), revealed that all species share conserved sequences to that of *Boa constrictor* chromosomes, particularly with the macrochromosomes pairs 1, 4, 7 (Figs. 5a–c, 6a–d). The cross-species hybridization (*Boa constrictor*/*Bothrops bilineatus*) revealed shared sequences on the centromeric position of the 4th and 7th pairs (Fig. 5a). However, the *Boa constrictor*/*Bothrops taeniatus* pitviper comparisons showed hybridization signals only on the centromeric position of the 4th pair (Fig. 5b). The *Boa constrictor*/*Bothrops atrox*—amazonian pitviper comparisons, on the other hand, showed hybridization signals only on the centromeric position of the 7th pair (Fig. 5c).

Comparisons between *Boa constrictor*/*Lachesis muta* revealed shared hybridization signals on the 1st and 7th chromosomal pairs. However, *Lachesis* hybridization signals on the 1st pair were more intense than *Boa constrictor* signals (Fig. 6a). The *Boa constrictor*/*Crotalus terrificus* showed shared sequences on the 4th and 7th pairs, similar to that of the *Boa constrictor*/*Bothrops bilineatus* (Fig. 6b). The *Boa constrictor*/*Crotalus ruruima* comparisons showed the same hybridization pattern to that of *Boa* / *Lachesis*, with signals only on the 1st and 7th pairs, likewise with more intense signals on the 1st pair (Fig. 6c). Unlike most patterns, *Boa constrictor*/*Spilotes pullatus* showed hybridization signals on 3 chromosomal pairs: near the pericentromeric region of the 1st pair (similar to that of the *Boa* / *Lachesis *and *Boa*/*C. ruruima*) and on the centromere of both 4th and 7th pairs (similar to those of the *Boa*/*B. bilineatus* and *Boa*/*C. terrificus*) (Fig. 6d). The 2nd pair was the sole representative with strong *Boa constrictor* specific hybridization signals, but with no shared regions of gDNA with all caenophidian snakes.

For all interspecific comparisons (Fig. 4a,b), we used pooled male and female gDNA from the seven species of caenophidian snakes (advanced lineages ZW) against chromosome spreads of *Boa constrictor* male and female. Once the hybridization patterns from all Caenophidian snakes were exactly the same on the chromosomes of red-tailed boa males and females, for convenience, representative metaphase was selected for illustrating the above results among the genomic Henophidia and Caenophidian comparisons.

### BAC mapping on *Boa constrictor* chromosomes

**Figure 7:**
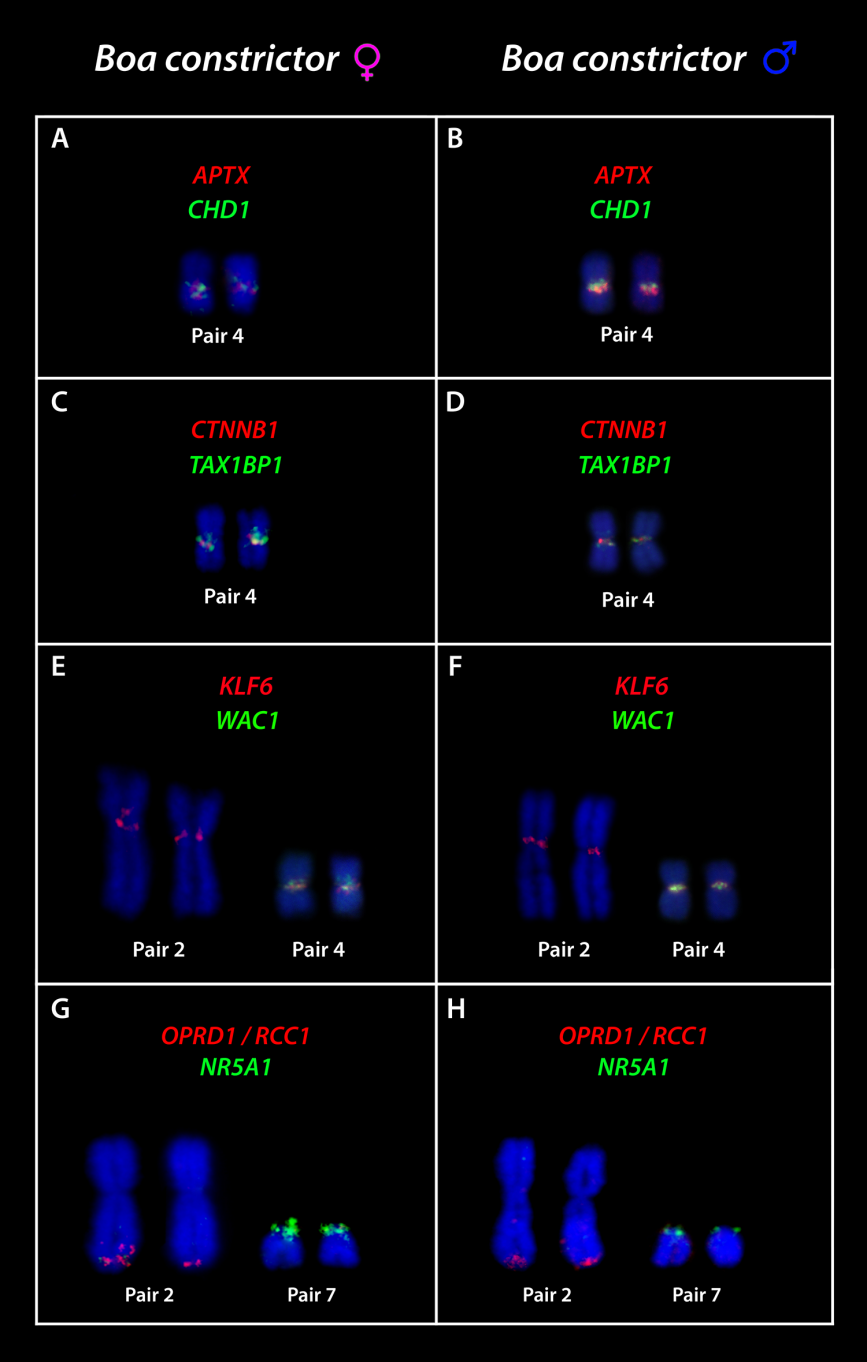
The mapping of eight BAC genes on the *Boa constrictor* male and female, showing marking mainly in the 2nd and in the 4th pairs. ISIS software was used for microphotography and analyzing images.

All *Pogona vitticeps* derived BACs used showed hybridized signals on *B. constrictor* chromosomes (Fig. 7a–h). Six BAC clones (*APTX*; *CHD1*; *CTNNB1*; *TAX1BP1*; *KLF6*; *WAC1*) were mapped to the centromeric position of the 4th pair of *Boa constrictor*, however, the *KLF6* was also mapped on the centromeric position of the 2nd pair. The *3L7* and *NR5A1* were mapped to the terminal position of the 2nd pair and in the centromeric position of the 7th pair respectively. The *ATPX* and *CHD1* are located on the 2nd pair of *Pogona vitticeps*, *CTNNB1*; *TAX1BP*; *KLF6*; *WAC1* on the 6th pair whereas *OPRD1/RCC1* and *NR5A1* on the sex chromosomes^19,52,53^.

## Discussion

**Figure 8:**
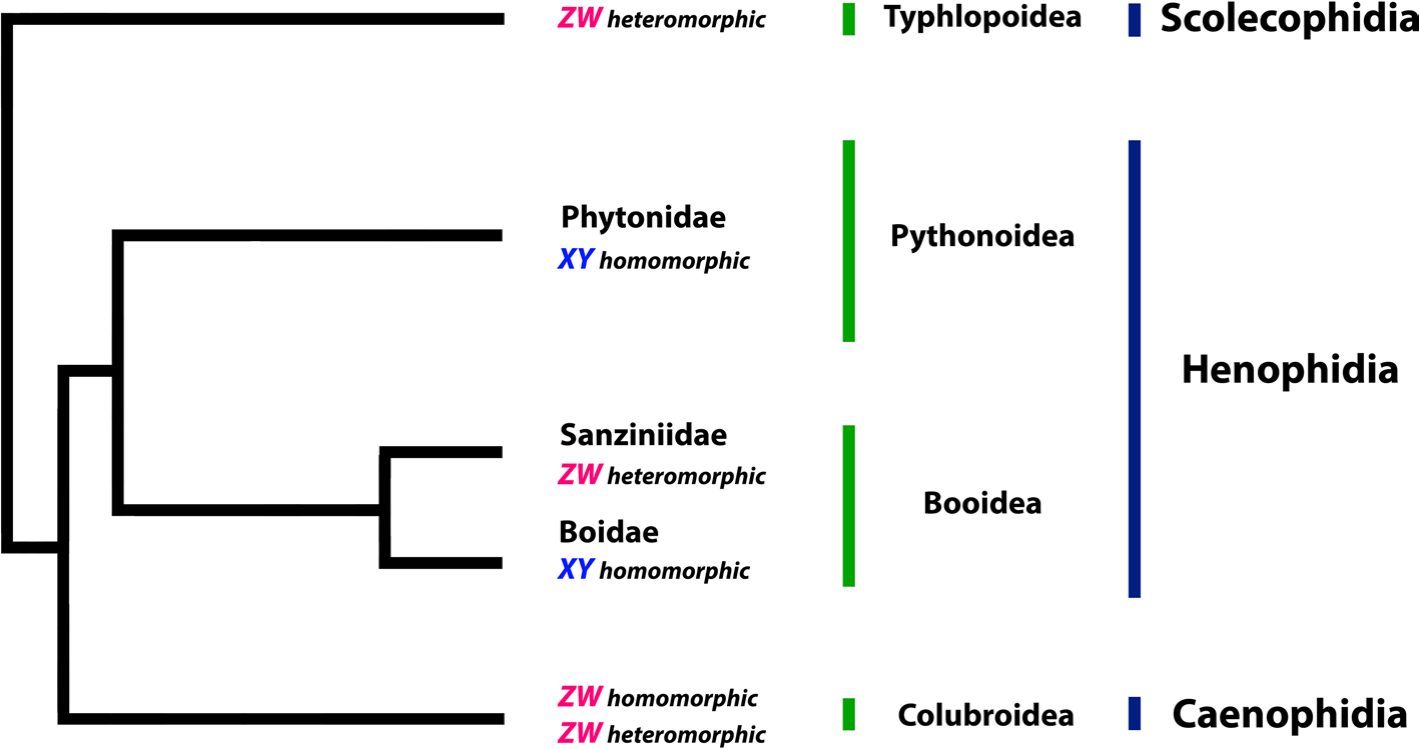
Relationships among the four major extant groups of snakes. Highlighting the transitions among homomorphic and heteromorphic ZW-XY-ZW systems across evolution. Phylogenetic tree adapted from Pyron et al.^73^ and Reynolds et al.^74^.

In reptiles, independent turnovers and transitions among sex chromosomes systems (XY and ZW) and sex-determining mechanisms (TSD and GSD) within closely related species are more common than previously thought, being thus, a widespread feature among non-avian reptiles^1,3,4,54,55^. Unlike most snakes, *Boa imperator* was reported to have XY homomorphic sex chromosomes^26^, and its sister species *Boa constrictor*, shares ancestry presenting also an XY system. Therefore, it is plausible that transitions between homomorphic ZW and XY have occurred in the Boidae family without much substantial genotypic innovation (e.g. considering the 4th pair of boas as the putative sex chromosomes, the XY and ZW are morphologically similar), as reported in the Japanese frog *Glandirana rugosa*^56^. In our study, we did not detect any sex-specific pattern using intra- and interspecific CGH experiments (Figs. 3, 5, 6), suggesting that only minute sequence differences exist between sex chromosomes (putatively the pair 4). A similar pattern was observed in the Sanziniidae family, a sister group to Boidae (Fig. 8)^32^, but the Z and W chromosomes of *Acrantophis* sp. cf. *dumerili* are morphologically well-differentiated nevertheless.

Whilst CGH has been applied for the identification of undifferentiated or cryptic sex chromosomes across a range of vertebrates ranging from fish to reptiles^45,46,50,51,57,58^, this technique, in some cases, may not be efficient in detecting specific sex domains (even in the heteromorphic sex chromosomes) as already seen in amphibians^59^ and in the well-differentiated ZW present in *Acrantophis* sp. cf. *dumerili* (Booidea)^32^. Perhaps some ancestral lineages still need more time to achieve sex-specific signatures (e.g. morphological changes, accumulation of sequences, heterochromatinization), or simply use alternative mechanisms for sex chromosome evolution, which makes it difficult to detect, especially when they retain huge traits of homology, as here observed in *Boa constrictor*.

**Figure 9:**
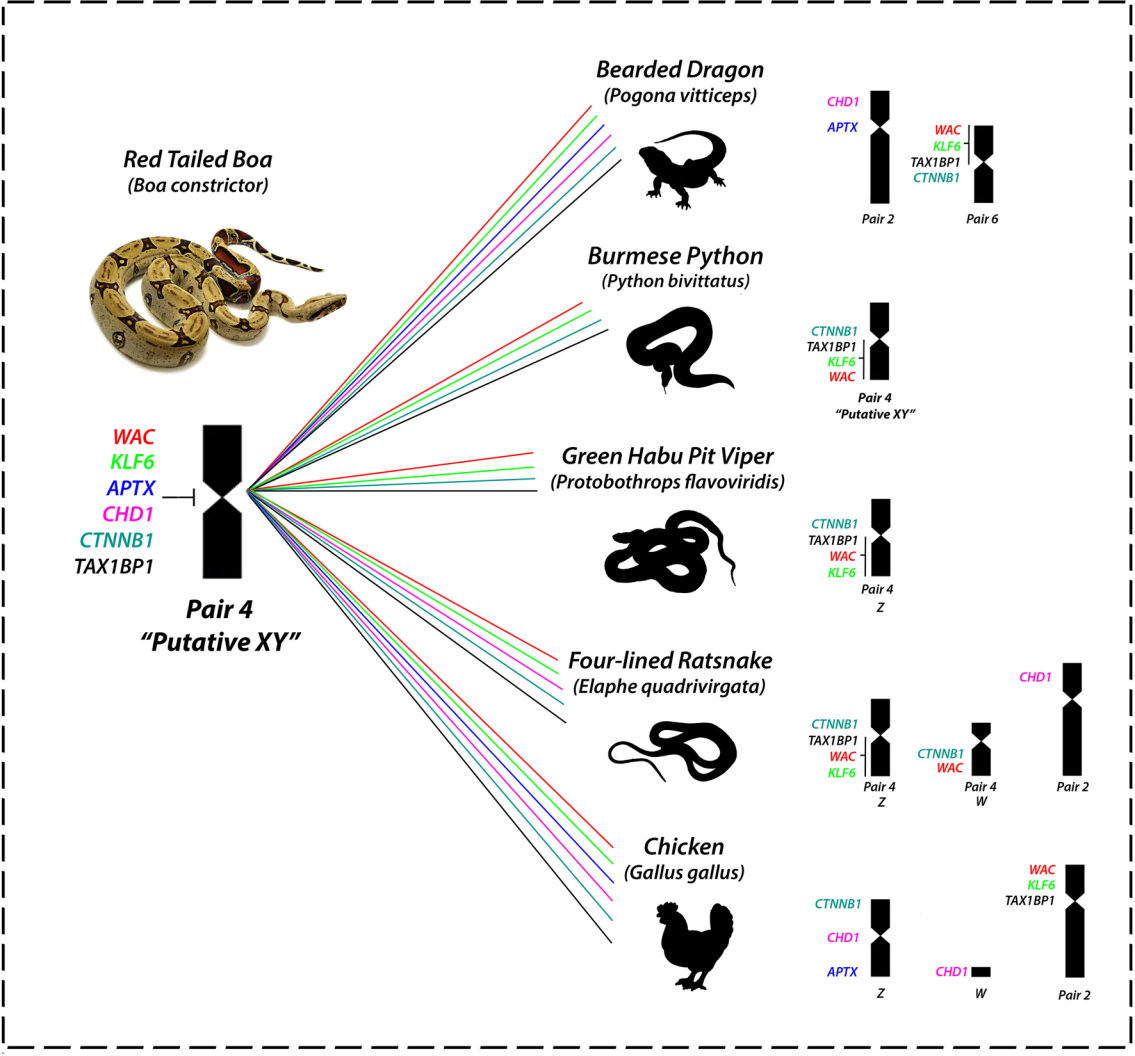
Comparison of six BACs located on the 4th pair of *Boa constrictor* with lizard, snakes and chicken. The mapping of BACs besides of identify homology with autosomal and sex chromosomes also indicate that gene repositioning occurred along evolution of these species.

Our comparative cytogenetic analysis suggests that henophidian and caenophidians indeed followed different evolutionary pathways regarding the origin of their sex chromosomes. Several genes share ancestry between putative homomorphic X and Y chromosomes of *Python* (at that time considered to be Z and W) and the Z chromosomes of caenophidians^27^, suggesting that X, Y and Z chromosomes can easily undergo transitions in ancestral lineages conferred by the similarity of morphology and gene content. For instance, even though located in different positions regarding other snakes’ lineages, the genes linked to the putative sex pair of *Boa constrictor* male and female points homology with the independently evolved putative pair of burmese python (*Python bivitattus* XY), with the sex pair of habu pit viper (*Protobothrops flavoriridis* ZW) and the four-lined ratsnake (*Elaphe quadrivirgata* ZW) (Fig. 9). Interestingly but not surprisingly, once sex chromosomes evolve fastly and independently across lineages^4^, the XY of *Python bivittatus* seems to share more similarities with caenophidians than with the other sole representative XY system existing in Serpentes, the XY present in *Boa*^26^. Regardless, this shared ancestry, in spite of some fine adjustments in the gene position on the sex pair, indicates that henophidian and caenophidian snakes do not share the same set of sex-determining genes, since other genes located on the putative XY of *Boa* also share homology with the second pair of *Elaphe quadrivirgata* (Caenophidia), which partially correspond to the Z chromosome of chicken. Furthermore, the mapping of *BaW* chromosome probe also provided strong evidence that caenophidian and henophidian snakes do not share the same sex chromosomes, because the W of *Bothrops atrox* (Caenophidia) has homology with the 7th autosomal pair of *Boa constrictor* and not with the putative homomorphic sex chromosomes (4th pair) (Fig. 1), which correspond to a well-differentiated ZW system in the sister group (Sanziniidae) ^32^. CGH also revealed that, among all the 7 caenophidians snakes involved in our comparative study, six of them shared ancestry with the 7th pair of *Boa constrictor*, that somehow share some degree of homology with the W sex chromosome of *B. atrox*. Perhaps this 7th pair represent a large conserved segment of the henophidians and caenophidians ancestor. To fully understand the real status of ZW–XY–ZW transitions and homology of sequences, combined whole genome sequencing and refined cytogenetic approaches will be required, especially in representatives from the four major clades of Serpentes suborder (Typhlopoidea ZW, Pythonoidea XY, Booidea ZW/XY, and Colubroidea ZW) (Fig. 8), where the XY sex chromosome system arose only twice and remained morphologically undifferentiated.

**Figure 10:**
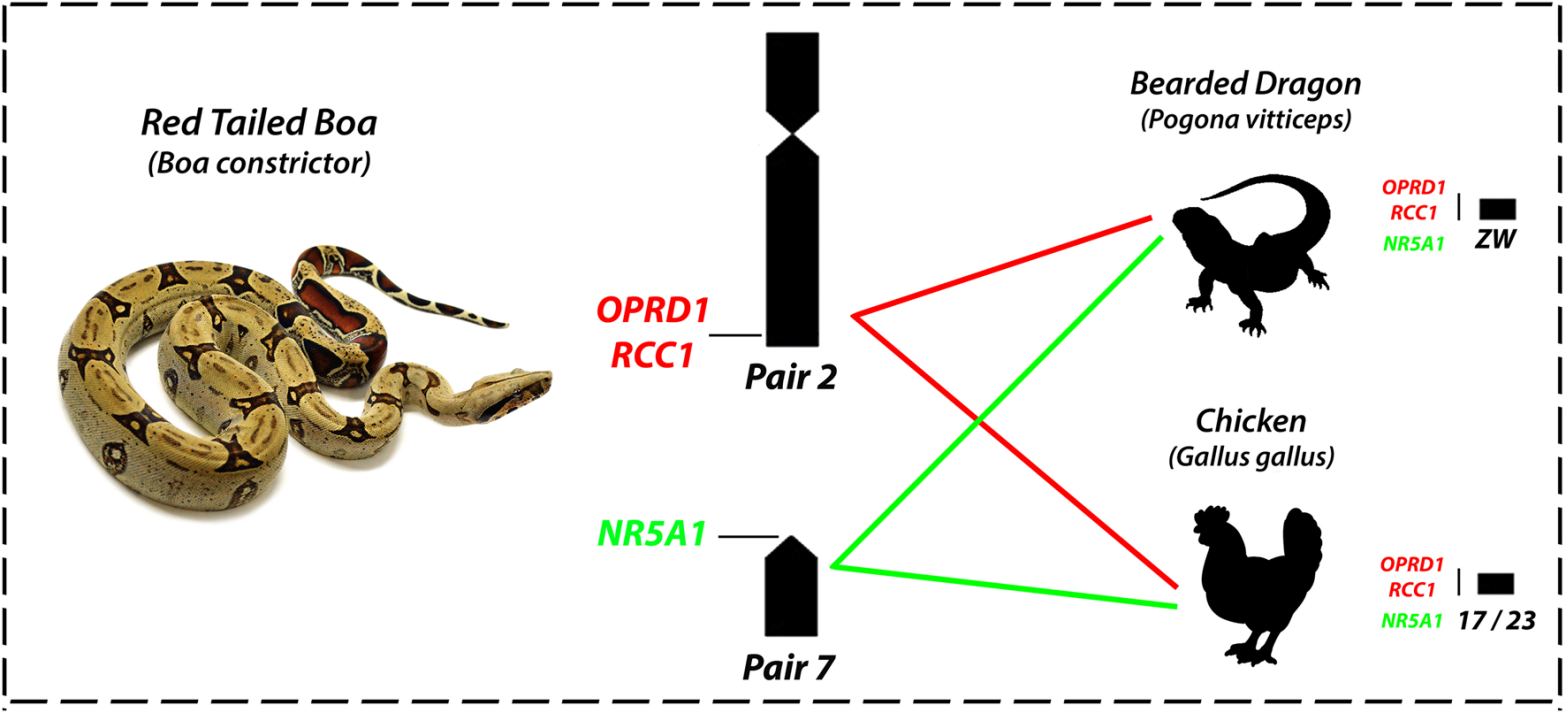
Comparative mapping of two sex linked genes in the bearded dragon (*Pogona vitticeps*) with *Boa constrictor* and chicken chromosomes.

Notably, our study revealed that the 4th pair of *Boa* also shares homology with the Z, W and 2nd chromosome pair of chicken (Fig. 9). While chicken’s Z partially correspond to the Squamata chromosome 2^2,29,60^, however, at least 2 genes (*CHD1*, *APTX*) located on the 4th pair of *Boa constrictor* also share homology with the second pair of *Elaphe quadrivirgata* and the bearded dragon (*Pogona vitticeps*). As hypothesized by Ezaz and colleagues, this synteny among different squamate clades and chicken Z chromosome could represent part of an ancestral super-sex chromosome for Aminiotes^2^. Interestingly, two sex-linked genes in *Pogona vitticeps* also share homology with the *Boa constrictor* 2 (BAC containing genes *OPRD1* and *RCC1* ) and 7 (*NR5A2*) chromosome pairs (Fig. 10). These genes correspond to the chicken chromosomes 17 and 23^19^. Concordantly, *OPRD1* / *RCC1* and *NR5A2* genes, also mapped in the yellow and green anacondas (*Eunectes notaeus* and *Eunectes murinus*), cerrado rainbow boa (*Epicrates crassus*) and in the amazonian puffer snake (*Spilotes pullatus*), showed a similar scenario (Viana personal communication). Although the homology of Squamates 2 and chicken Z is considered a conserved trait across lineages^2,12,29, 61–63,76^, the *Boa constrictor* 2 shares homology to the chicken chromosome 17 and 23, whereas the chicken Z, W and 2 with the putative XY of amazonian red-tailed boa (4th pair) (Figs. 9, 10), which highlights the homology and ancestry of sequences among close and distantly related lineages, possibly remnants of a common evolutionary history among avian and non-avian reptiles.

**Figure 11:**
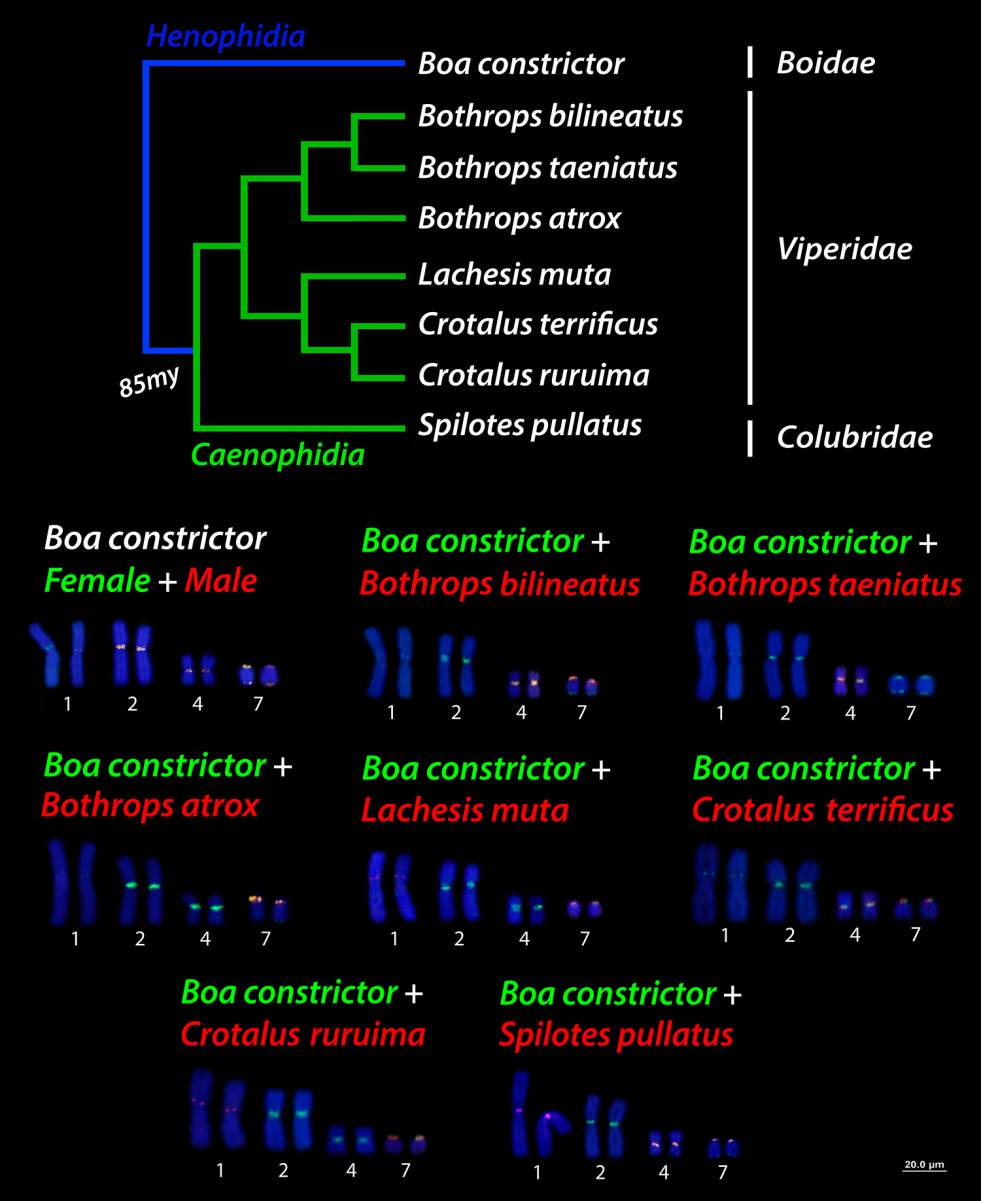
Relationships among Henophidia and Caenophidia species highlighting the reciprocal mapping male and female gDNA of Caenophidia species in *Bc* chromosomes. The phylogenetic tree was adapted from Figueroa et al.^75^.

It is intriguing that after the divergence of Henophidia and Caenophidia in the Upper Cretaceous (~ 85 MYR)^64,65^ snakes still share conserved sequences across lineages even after such long period of independent evolution (Fig. 11). Even more puzzling is that some closely related lineages (e.g. *C. terrificus* and *C. ruruima*) show a divergent pattern of gDNA hybridization on the *Boa constrictor* chromosomes (Figs. 6b,c, 11), perhaps unique particularities at species level. For the two rattle snakes, only *C. terrificus* shared sequences on the putative homomorphic sex chromosomes of *Boa constrictor*. Likewise, the *Bothrops* species (*B. bilineatus*, *B. taeniatus* and *B. atrox*), showed divergent patterns of hybridization to *Boa constrictor* chromosomes (Figs. 5a–c, 11). This evolutionary landscape might be product of the mechanisms that shape the processes of chromosomal differentiation during the evolution, as for example the association with TEs (Transposable Elements) and SSRs (Simple Short Repeats) sequences, that triggers an important role on the genome architecture leading to independent evolution processes (e.g. silencing, deleting, or increasing genomic regions)^63,66–68^. This seems to be also the case for the snakes here analyzed.

Nevertheless, all caenophidians used in our study shared sequences with *B. constrictor* chromosomes, representing a possible inheritance of ancestry, being the assortment of hybridization patterns due to the *tempo* of sequence divergence and transient evolutionary mechanisms linked to their evolution spanning ~ 85 my of independent evolution. However, we were not able to identify any sex-specific sequence from all caenophidians gDNA derived probes and W chromosome probes (*BaW*), that somehow showed the same hybridization pattern in both male and females of *Boa constrictor*. In fact, this is not surprising because hybridization has not even detected within *Boa* comparisons, such sex-specific patterns. The shared sequences and different patterns could simply be the result of the convergent accumulation of repetitive sequences during Snakes’ evolution. However, all caenophidian species used here share the same W sex chromosome (Viana personal communication).

This lack of sex-specific signals in *Boa* (XY) from caenophidian (ZW) gDNA derived probe is likely that the sex-linked sequences in advanced snakes are different and, therefore, do not share any similarity with those sex-linked sequences in *Boa*. This suggests an independent evolution of sex chromosome sequences in snakes, but in caveats, given the similarity of morphologies and gene content of putative sex pair of henophidian and the sex chromosomes of caenophidian snakes we cannot conclusively infer which homomorphic system, XY or ZW really occurs in ancestral lineages (Boidae and Pythonidae). Regardless, our study provides first evidence that caenophidian and henophidian snakes have a common evolutionary history but likely evolving a different set of sex-determining sequences, where the sex chromosomes followed divergent evolutionary pathways. However, in henophidians, the real status of homology with the cryptic sex chromosomes of *Boa* and the heteromorphic ZW present in the sister group (Sanziniidae) is yet to be investigated, which will require developing probes from Y sex-linked markers of *Boa imperator* for cross species chromosome mapping. Such combined methods of genomics and cytogenetics will enable us to unreveal the dynamic evolutionary history and transitions between XY and ZW sex chromosomes system in the major clades of Serpentes. This study is part of a series of further cytogenetic and genomic studies, focusing on Neotropical reptiles and their hidden evolutionary diversity.

## Material and methods

### Sampling, mitotic chromosomes preparation, and DNA extraction

Snakes were collected from natural populations across Amazon region under permission granted by Instituto Chico Mendes de Conservação da Biodiversidade (ICMBio) number 45275. We analyzed chromosomes of six males and seven females of *Boa constrictor* and the genomic DNA (gDNA) of several caenophidian snakes with differentiated ZW chromosomes (*Bothrops bilineatus*, *B. taeniatus*, *B. atrox*, *Lachesis muta*, *Crotalus terrificus*, *C. ruruima* and *Spilotes pullatus*) in the cross-species mapping. Chromosomal preparations were obtained following^69^. The gDNA of males and females for all species were extracted from blood using the Wizard Genomic Purification Kit (Promega), according to the manufacturer’s recommendations. We also highlight that in our present study, no animal needed to be euthanized.

### Microdissection of W sex chromosome of *Bothrops atrox* and preparation of the *BaW* chromosome paints

We performed microdissection using an inverted phase-contrast microscope Zeiss Axiovert.A1 (Zeiss, Oberkochen, Germany) equipped with Eppendorf TransferMan NK 2 micromanipulator (Eppendorf, Hamburg, Germany). We prepared glass needles from 1.0 mm diameter capillary glass using a glass capillary puller, Sutter P-30 Micropipette Puller (Sutter Instrument, Novato, Calif., USA) and sterilized using ultraviolet irradiation. We microdissected a W chromosome from freshly prepared slides of a female *B. atrox* using a glass needle and the micromanipulation system, subsequently transferring the W chromosome into 0.2 ml PCR tubes. The W chromosome DNA (*BaW*) was amplified using GenomePlex Single Cell Whole Genome Amplification Kit (Sigma-Aldrich, St. Louis, Mo., USA) according to the manufacturer’s protocol with slight modifications according to^70^. The volume of the reactions was scaled down to half, and the PCR amplification step was increased to 30 cycles. The W chromosome paint of *B. atrox* was labeled by nick translation means incorporating SpectrumGreen-dUTP (Abbott, North Chicago, Ill., USA). The hybridization was carried out for 1 day in the *B. atrox* chromosomes (control) and 3 days in cross-species chromosome painting (*Boa constrictor* male and female).

### Preparation of probes for CGH

The gDNA of males and females of all species was used for comparative approaches focused on an intraspecific comparison between males and females of ***Bc*** (*Boa constrictor*), with special emphasis on the homomorphic sex chromosomes in this species and in an interspecific genomic comparison among henophidian and caenophidian species. For intraspecific comparisons, male and female-derived gDNA of *Boa constrictor* were hybridized against male and female metaphase chromosomes of the species (Fig. 2). The female-derived gDNA was labeled with biotin-16-dUTP and male gDNAs with digoxigenin-11-dUTP by Nick translation means (Roche, Mannheim, Germany). Interspecific comparisons gDNA of male and female of all caenophidian species were hybridized against metaphase chromosomes and gDNA of male and female of *Boa constrictor* (***Bc***) (Fig. 4a,b). For this purpose, the gDNA of caenophidians male and female (green pit viper **/** *Bothrops bilineatus* = ***Bb***; forest pit viper *Bothrops taeniatus* = ***Bt***; amazonian pit viper / *Bothrops atrox* = ***Ba***; bushmaster/*Lachesis muta* = ***Lm***; common rattle snake/*Crotalus terrificus* = ***Ct***; north rattle snake/*Crotalus ruruima* = ***Cr*** and puffer snake/*Spilotes pullatus* = ***Sp***) were labeled with digoxigenin-11-dUTP (red), whereas male and female-derived gDNA of *Boa constrictor* (***Bc***) were labeled with biotin-16-dUTP (green) by Nick translation means above mentioned. For both intra and interspecific purposes, the final hybridization mixture for each slide was composed of gDNAs of the species (500 ng each), 20 μg of male-derived C_0_t-1 DNA (i.e. fraction of genomic DNA enriched for highly and moderately repetitive sequences, prepared according to^71^) and 20 μl of the hybridization buffer containing 50% formamide, 2 × SSC, 10% SDS, 10% dextran sulfate and Denhardt´s solution, pH 7.0.

### FISH used for CGH

The FISH experiments were performed primarily according to^72^ and subtle modifications according to our previous studies. The slides were incubated at 37 °C in a dark humid chamber for three days and the hybridization signals were detected with Anti-digoxigenin-Rhodamin (Roche) diluted in 0.5% bovine serum albumin (BSA) in phosphate-buffered saline (PBS), and avidin-FITC (fluorescein isothiocyanate, Sigma) diluted in PBS containing 10% normal goat serum (NGS). The chromosomes were counterstained with DAPI (1.2 µg/ml) and mounted in an antifade solution (Vector, Burlingame, CA, USA).

### Bacterial artificial chromosome (BAC) preparation and FISH

We mapped *Pogona vitticeps* derived 8 BAC clones containing 8 chromosome-linked genes (*WAC*; *KLF6*; *APTX*; *CHD1*; *CTNNB1*; *TAX1BP1; OPRD1/RCC1* and *NR5A1*)^12,19,52,53^ to *B. constrictor* male and female metaphase chromosomes. The clones were selected from *Pogona vitticeps* genomic BAC library as previously described in Ezaz et al.^12,52^, Young et al.^53^ and Deakin et al.^19^. All 8 BACs were anchored to *P. vitticeps* metaphase chromosomes as control (data not shown). BAC DNA was extracted using the Promega Wizard Plus SV Minipreps DNA Purification System following the manufacturer’s protocol, with volumes scaled up for 15 ml cultures. The BACs were labeled with SpectrumOrange-dUTP or SpectrumGreen-dUTP (Abbott, North Chicago, Ill., USA) and hybridized for 2 days. The slides were then washed twice in 0.4 × SSC, 0.3% IGEPAL (Sigma-Aldrich) at 55 °C for 5 min each and after air-dried, counterstained using DAPI (1.2 µg/ml) and mounted in an antifade solution (Vector, Burlingame, CA, USA).

### Microscopy and image analyses

Images were captured using an Olympus BX51 microscope (Olympus Corporation, Ishikawa, Japan) with CoolSNAP. For W painting and BAC-FISH, images were captured using a Zeiss Axioplan epifluorescence microscope equipped with a CCD camera (Zeiss). ISIS software was used for microphotography and analyzing images.

### Ethics statement

We declare that all procedures and experimental protocols were approved and performed under the rules of the Ethics Committee of the National Institute of Amazonian Research (Permission number: 018/2017).
